# 

**Published:** 1942

**Authors:** 


### The Medical Library of the University of Bristol

WITH WHICH IS INCORPORATED

**The Library of the Bristol Medico-Chirurgical Society**

*The following donations have been received since the publication of the list in the last issue :—*

| | |
|---|---|
| Dr. J. S. Baxter (1) .. .. .. .. .. | 1 volume. |
| Professor R. J. A. Berry (2) .. .. .. .. | 1 volume. |
| The late Professor Fawcett (3) .. .. .. .. | 2 volumes. |

| | |
|---|---|
| General Medical Council (4) | 1 volume. |
| Messrs. Wm. Heinemann (Publishers) (5) | 1 volume. |
| The Henry Phipps Institute (6) | 1 volume. |
| Professor E. W. Hey Groves (7) | 7 volumes. |
| Munro Smith Fund (8) | 1 volume. |
| Professor J. A. Nixon (9) | 1 volume. |
| Dr. G. S. Parkinson (10) | 1 volume. |
| W. H. Ross Foundation (Scotland) (11) | 1 volume. |
| J. E. Shaw Bequest (12) | 2 volumes. |
| Smithsonian Institute (13) | 1 volume. |
| Messrs. John Wright & Sons Ltd. (14) | 4 volumes. |

Unbound periodicals have also been received from Dr. S. B. Adams, Dr. R. F. Barbour, Professor E. W. Hey Groves, Dr. T. Milling, Professor C. Bruce Perry, Dr. J. Odery Symes, Messrs. John Wright & Sons Ltd.

### THE ONE HUNDRED AND SEVENTY-SIXTH LIST OF BOOKS.

The figures in round brackets refer to the figures after the names of the donors. The books to which no such figures have been attached have either been bought from the Library Fund or received through the *Journal*.

| | | | |
|---|---|---|---|
| *Album XVI.* International Medical Congress | | (3) | 1909 |
| **Bailey, Hamilton** (Ed.) | *Pye's Surgical Handicraft* | 13th Ed. (7) | 1942 |
| **Bailey, Hamilton** | *Demonstrations of Physical Signs in Clinical Surgery* | 8th Ed. (7) | 1942 |
| **Bailey, Hamilton** | *Surgery of Modern Warfare.* Vol. I. | 2nd Ed. (7) | 1942 |
| | *Surgery of Modern Warfare.* Vo. II. | 2nd Ed. | 1942 |
| **Baxter, Dr. J. S.** | *Aids to Surgical Anatomy* | 2nd Ed. (1) | 1942 |
| **Berkeley, A. Comyns** | *Handbook of Midwifery* | 11th Ed. | 1941 |
| **Berry, R. J. A.** | *Your Brain and its Story* | (2) | 1939 |
| **Bertwistle, A. P.** | *Descriptive Atlas of Radiographs* | 5th Ed. (14) | 1942 |
| **Bramwell, C., and King, J. T.** | *Principles and Practice of Cardiology* | | 1942 |
| *British Encyclopædia of Medical Practice*— | | | |
| | Surveys and Abstracts | | 1941-2 |
| | Cumulative Supplement | (12) | 1941-2 |
| *British Pharmacopœia*, 1932— | | | |
| | Second Addendum | | 1940 |
| | Fourth Addendum | | 1941 |
| **Brockbank, E. M.** | *Mule Spinners' Cancer* | | 1941 |

| | | | |
|---|---|---|---|
| **Clay, H. H.** .. .. .. | *Sanitary Inspectors' Handbook* .. | 5th Ed. | 1942 |
| **Conybeare, J. J.** .. .. | *Textbook of Medicine* .. .. .. .. .. .. .. | | 1942 |
| **Davies, H. M., and Coope, R.** (Eds.) | *War Injuries of the Chest* | .. (14) | 1942 |
| **Dilling, W. J., and Hallam, S.** | *Dental Materia Medica* .. | 2nd Ed. | 1941 |
| **Gruner, O. C.** .. .. .. .. | *A Study of the Blood in Cancer* .. | .. (14) | 1942 |
| **Harries, E. H. R., and Mitman, M.** | *Clinical Practice in Infectious Diseases* | | 1940 |
| **Hunter, W.** .. .. .. | *Human Gravid Uterus.* (Sydenham Society).. | | 1851 |
| **Jameson, W. W., and Parkinson, G. S.** | *Synopsis of Hygiene* .. | 7th Ed. (10) | 1942 |
| **Key, J. A., and Conwell, H. E.** | *The Management of Fractures, Dislocations and Sprains* .. .. .. | 3rd Ed. (7) | 1942 |
| *Lewis's Library Catalogue.* | Supplement, 1938–41 .. .. .. .. .. .. .. | | 1942 |
| **Linell, E. A.** .. .. .. | *Distribution of Nerves in the Upper Limb* | (3) | 1920 |
| **Macleod, J. J. R.** .. .. | *Physiology in Modern Medicine* | 9th Ed. (8) | 1942 |
| **Maximow, A. A., and Bloom, W.** | *Textbook of Histology* .. .. | 4th Ed. | 1942 |
| **Mayo, Katherine** .. .. | Volume II. .. .. .. .. .. .. .. .. | (9) | 1931 |
| **McMeeken, M.** .. .. .. | *Developmental Aphasia in Educationally Retarded Children.* [W. H. Ross Foundation (Scotland)] .. .. .. .. .. .. .. .. | (11) | 1942 |
| Medical Research Council. | Special Report Series, 243. *Chronic Pulmonary Disease in South Wales Coalminers.* | | |
| Medical Research Council. | Special Report Series, 245. *Report of the Committee on Bed-bug Infestation* .. .. .. | | 1935–40 |
| **Middlesex Hospital** .. .. | *Collected Papers in Physiology and Medicine* | (7) | 1941 |
| **Pavlov, I. P.** .. .. .. | *Conditioned Reflexes.* Vol. II. .. .. .. .. .. | | 1941 |
| **Piney, A.** .. .. .. .. | *Synopsis of Blood Diseases* .. .. | (5) | 1942 |
| **Scott, S. G.** .. .. .. | *Monograph on Adolescent Spondylitis* .. .. | | 1942 |
| **Sherman, H. C.** .. .. | *Chemistry of Food and Nutrition* | 6th Ed. | 1941 |
| **Sigerist, H. E.** .. .. .. | *Man and Medicine* .. .. .. .. .. .. .. | | 1932 |
| **Thompson, C. J. S.** .. | *History of Surgical Instruments*.. .. .. | (7) | |
| **Wallace, A. B.** .. .. | *The Treatment of Burns* .. .. .. .. .. .. | | 1941 |

### TRANSACTIONS, REPORTS, JOURNALS, ETC.

| | | |
|---|---|---|
| Annual Report of the Smithsonian Institute .. .. .. .. .. .. | (13) | 1940–41 |
| Henry Phipps Institute (University of Pennsylvania, 29th Report | (6) | 1940–41 |
| The Medical Annual .. .. .. .. .. .. .. .. .. .. .. .. | | 1942 |
| The Medical Register .. .. .. .. .. .. .. .. .. .. .. | (4) | 1942 |
| Quarterly Cumulative Index Medicus. Vol. 30. July—December .. .. | | 1941 |

### Publications Received

From Messrs. H. K. Lewis & Co. Ltd.:

Catalogue of Lewis's Medical and Scientific Lending Library. Supplement, 1938–41. Price 4s.

*Instructions for Measuring Insulin.* By S. L. Simpson. Price 8d. per card.

From Oxford University Press:

*Cancer of the Uterus.* By Elizabeth Hurdon. Price 17s. 6d.

*Oxford War Manual: Amputations and Artificial Limbs.* By R. D. Langdale-Kelham, M.R.C.S., L.R.C.P., and George Perkins, *M.C.*, F.R.C.S. Price 5s.

From John Wright & Sons Ltd.:

*British Journal of Surgery.* No. 117. Vol. 30. Price 12s. 6d.

*Pye's Surgical Handicraft.* 13th Edition. Edited by Hamilton Bailey, F.R.C.S. Price 25s.

*Tuberculosis in Childhood.* By Dorothy S. Price. Price 17s. 6d.

### Local Medical Notes

Two students of the University have been elected to Rockefeller Medical Studentships:—

Susanna Foss, at University of Chicago.
Grace V. Andrew, at Tulane University.

EXAMINATION RESULTS.

**University of Bristol.**—Students of the University have recently passed the following examinations:—

M.B., Ch.B.—*First Examination.* Margaret J. Andrews, L. W. J. Clarke, W. L. Codd, G. J. S. Herdman, Una P. Jones, Mary Knowles, G. E. Mitchelmore, Jean E. Morrell, J. P. Norris, S. M. Stotesbury, Sybil M. Tuffin. *In Biology:* F. G. Bolton.† *In Physics and Organic Chemistry:* D. H. Drummond.† *Supplementary First Examination:* Endora M. R. Davies, C. E. Halliday, Lisa E. Mandelbaum, P. J. D. Benjafield, D. M. Cunningham, G. Edwards, M. E. Hincks, Catherine M. Hoyle, Jean Musgrave, J. S. R. R. Old, F. A. A. Ruggeri.

M.B., Ch.B.—*Second Examination.* M. Alms, Grace V. Andrew, J. B. Brierley, Elizabeth Bryant, N. J. Cook, R. W. Drewer, J. L. Dunscombe, A. C. Edwards, J. G. R. Ellis, E. W. Emery, Lorna M.

Green, Irene D. R. Gregory, J. R. Hawkings, D. H. Johnson, Margaret A. Lamb, Edith J. Lowick, Margaret M. Moncrieff, R. A. J. Pearce, Dorothy M. Reece, Patricia M. Rundle, R. H. Scott, Barbara F. Smith, S. A. K. Stokes, T. Straton, Edith E. Thomson, A. D. Verniquet, W. Wagland, Pamela Westhead. *Group I only:* P. J. Chapman, Margaret J. Cox, Pamela M. Farmer, Sheila E. Fraser, D. Purnell, A. D. Stewart. *Group II:* J. D. Medhurst,† A. D. Stewart.†

M.B., CH.B.—*Final Examination* (*Section I*)*:* Janet Baker, J. E. Beviss, J. H. Challenger, A. G. M. Davies, C. H. Gurd, Mercia J. Griffiths, Beryl M. Hinckley, Elizabeth Hodson, J. E. G. Hope-Scott, W. G. H. Hunt, D. E. Olliff, A. P. Radford, Rukmini L. Reddy, D. A. Sarsfield, Margaret Savile, A. J. F. Saxton, S. S. Short, T. R. Stephens, I. F. R. Sutherland.

M.B., CH.B.—*Final Examination.* Nancy R. Baldwin (1), R. F. Brooks, J. H. Challenger, Rosalie M. Child, D. G. Davies, J. Deighton, Mary D. Dixon, G. F. M. Hall, Joyce Kennard, (Mrs.) J. Kerr, A. G. H. Mitchinson (2), E. W. Moore, J. L. Pardoe, A. F. Rogers, A. R. Rowe, (Mrs). M. E. Seddon, (Mrs.) M. Shanks, N. Slade, I. F. R. Sutherland, M. H. Thomas, G. H. Valentine (3), Jean Wallace, G. A. Walton, G. H. Wattley (2, 4). *Group I only:* W. G. H. Hunt. *Group II:* K. L. Brown†, A. H. M. Oakes,† (Mrs.) D. Willoughby.

B.D.S.—*First Examination.* E. J. R. Morgan, W. Watson. *In Chemistry and Physics:* P. J. Sheldrick.

B.D.S.—*Supplementary First Examination.* P. J. Blyth.

B.D.S.—*Second Examination.* G. A. J. Comyn, A. N. R. Gibson, D. C. Sara, A. F. J. Smith.

B.D.S.—*Third Examination.* H. L. Leech, E. M. Pitt (5), J. R. V. B. Gibson.*† *Dental Anatomy only:* J. M. Allen, I. A. Morgan.†

B.D.S.—*Final Examination.* F. M. Warren. *Section I. only:* C. R. W. Birkett, J. R. V. B. Gibson (6), R. D. Hepburn, J. B. K. Mann.

L.D.S.—*First Examination.* A. D. Baxter.

L.D.S.—*Supplementary First Examination.* R. D. Jefferies, A. H. Kingham, J. P. Rees, A. Thomas.

L.D.S.—*Second Examination.* T. W. Beer, K. W. Bowrey, M. P. Hazell, J. C. Nicholas, Eileen Rich, J. F. V. Woolley.

L.D.S.—*Third Examination.* T. W. Beer,* A. R. Brook,*† C. Gibbs, Alice B. U. Leigh,*† Eileen Rich, W. H. E. Turnbull.*† *Dental Anatomy only:* J. W. B. Kay,† N. W. Leech,† L. H. Valentine.

L.D.S.—*Final Examination. Section II* (*Dental Surgery*)*:* W. D. Arnold,† Patricia M. Burke,† G. G. Davis,† G. Hellings,† D. A. Holmes,† I. E. Perry,† F. M. Warren.† *Section I:* W. E. C. Chaplin, H. B. Doubt, N. W. Leech, Alice B. U. Leigh, R. E. May, L. Murray, B. N. Neilson, B. A. Oliver, I. E. Perry, R. H. Shipway, R. C. Taylor, J. B. Westlake, J. B. Wood.

**University of Cambridge.**—M.B.—*Final Examination. Part I* (Surgery, Midwifery and Gynaecology): L. A. Scott White. *Part II* (Principles and Practice of Physic, Pathology and Pharmacology): (Mrs.) P. Eskell, D. S. Short.

**Society of Apothecaries, London.**—*Final Examination.* D. I. Fullerton (Midwifery),† A. F. Rogers (Obstetrics).†

**Royal College of Surgeons of England.**—*Primary Fellowship Examination.* J. B. Brierley.

**Conjoint Board.**—M.R.C.S., L.R.C.P.—*First Examination.* M. P. Bourke (7, 8), A. M. Brown (9, 10), Nina Dawson-Reid (9, 10), B. L. Finzel (7, 8), Sheila E. Fraser (10), G. A. K. M. Jahans (10), P. R. Miller (10), Phyllis D. Moses (7, 8), M. J. Mulcahy (10), Mildred I. Pott (7, 8), C. T. Ross (9, 10), A. D. Verniquet (9, 10).

M.R.C.S., L.R.C.P.—*Final Examination.* Margot H. Dickson (11), D. I. Fullerton† (12), F. J. Goddard† (12, 13), A. J. R. Hudson (11, 14), W. G. M. Hunt (11, 14), D. J. Jones (14), P. M. Keppich (14), A. F. Rogers (11, 12, 14), (Mrs.) M. Shanks (12), D. S. Short (11, 13), (Mrs.) D. M. Thorne (11, 12), G. H. Valentine† (14), E. L. Ward (14), G. H. Wattley† (14), J. I. Williams (12),† (Mrs.) D. Willoughby (13).

* In Dental Mechanics and the properties of materials used in Dental Practice.

† Completing the Examination.

(1) With Distinction in Surgery.
(2) With Distinction in Public Health.
(3) With Distinction in Obstetrics.
(4) With Distinction in Medicine.
(5) Distinction in Dental Anatomy.
(6) Distinction in Pathology.
(7) Pharmacology.
(8) Materia Medica.
(9) Anatomy.
(10) Physiology.
(11) Pathology.
(12) Midwifery.
(13) Medicine.
(14) Surgery.

---

As we go to press we learn with deep regret of the death of Emeritus Professor Francis Henry Edgeworth. An Obituary notice will appear in our next issue.

### Index

Anæsthesia, 19.

Anæsthesia for shocked patients, 74.

Bell, R. J. Irving.—The Bristol Scheme for dealing with an outbreak of Typhus, 16.

Books Reviews, 23, 71.

Bristol Scheme for dealing with an outbreak of Typhus Fever.—R. J. Irving Bell, 16.

Bristol University :—
Medical Library, 33, 79.
Examination Results, 39, 82.

Clarke, Colonel, return of, 73.

Experiences of Typhus Fever.—F. Kohn, 13.

Experimental Study of Development (Thirty-first Long Fox Lecture)—Prof. C. M. Yonge., 49.

Fundamental Principles of Fracture Treatment—K. H. Pridie, 7.

Future of Medical Practice, 29.

George Medal award to Squadron-Leader F. G. Mogg, R.A.F., 27.

Guirdham, A.—Inunction of Testosterone in Mental Disease, 1.

Hastings, Somerville.—Hospital re-organization, 61.

Hospital re-organization — Somerville Hastings, 61.

Inunction of Testosterone in Mental Disease.—A. Guirdham, 1.

Kohn, F.—Experiences of Typhus Fever, 13.

Long Fox Memorial Lecture (Thirty-first).—Experimental Study of Development.—Professor C. M. Yonge, 49.

Medical Mission Auxiliary, 27.

Mogg, Squadron-Leader F. G., R.A.F.—Award of George Medal to, 27.

Obituaries—
Edward Fawcett, 77.
C. A. Wigan, 78.
E. G. Hall, 79.

Pridie, K. H.—The Fundamental Principles of Fracture Treatment, 7.

Royal Naval Hospital, 73.

Story of the Bristol Hospitals, 66.

Typhus in Britain, 31.

Yonge, Prof. C. M.—The Experimental Study of Development (Thirty-first Long Fox Lecture), 49.

